# Does Changes in the Electrical Resistance of an Acupuncture Meridian Predict Pain Intensity Following Orthopedic Surgery?

**DOI:** 10.5812/aapm.7254

**Published:** 2013-03-26

**Authors:** Mehran Rezvani, Mahmoud-Reza Alebouyeh, Farnad Imani, Saeid Reza Entezary, Masood Mohseni

**Affiliations:** 1 Department of Anesthesiology, Isfahan University of Medical Sciences, Isfahan, Iran; 2 Department of Anesthesiology and Pain Medicine,Rasoul Akram Medical Center, Iran University of Medical Sciences (IUMS), Tehran, Iran

**Keywords:** Acupuncture, Pain, Electric Impedance

## Abstract

**Background:**

Several methods for assessment of severity of pain have been proposed but all of them are subjective.

**Objectives:**

This study evaluated the association concerning changes in electrical resistance (ER) between two acupuncture points and severity of postoperative pain in order to define an objective measurement of pain.

**Patients and Methods:**

In a cross-sectional study, 50 patients undergoing lower extremity orthopedic surgery with postoperative moderate to severe pain (VAS > 4,) were consecutively enrolled. In the recovery room, the patients' pain scores were assessed and in patients with VAS > 4, the electrical resistance between Li4 and Li11 acupuncture points as well as pain scores was measured prior and following analgesic administration.

**Results:**

Following meperidine use, the mean VAS significantly decreased and the ER between the two acupoints was significantly increased. However, Pearson correlation analysis did not reveal any association between the trends of pain intensity and ER (P > 0.05). The ER change in patients operated under epidural anesthesia was significantly less than those who experienced general or spinal anesthesia.

**Conclusions:**

There is a coincidence of pain relief and change in the ER of acupuncture meridians without significant association. The diagnostic value of ER for pain, stress response or any other physiologic outcome needs to be investigated in clinical trials with a well-defined control group, with more accurate instruments and probably in different acupuncture meridians.

## 1. Background

Pain is a subjective sense expressed by the patients and cannot be effortlessly measured by the clinicians (1). Available physiologic markers cannot reliably assess the pain severity, as well (2). Thus, several questionnaires and scales have been proposed to evaluate the patients' severity of pain including verbal rating scale, numerical rating scale and visual analogue scale (VAS) (3). These scales are valuable and widely used but have limited application when the patient is not cooperative such as in extremes of age, drug abuse, severe psychiatric disorders and loss of consciousness (4, 5). In these cases an objective measure of pain independent of the patients' cooperation is required. Acupuncture has a long history in the east and introduced to the western medicine in the 1950s (6). Since then, a number of studies have claimed that acupuncture points have their specific electrophysiologic properties and specifically reveal decreased electrical resistance (ER) (7-14). However, some earlier studies do not support this hypothesis (15, 16). Generally, it is believed that each organ is associated with its specific acupuncture points and meridians, and medical conditions influence the electrophysiologic properties of these points in different ways (17-21). The Li4 is a known acupuncture point generally used for pain relief usually with the attendance of Li11 or Li15 acupuncture points (22).

## 2. Objectives

This study was conducted to evaluate the association between changes in the ER between Li4 and Li11 acupuncture points and severity of postoperative pain in order to define an objective measure of pain.

## 3. Patients and Methods

### 3.1. Patients

In a cross-sectional study, a total of 50 American Society of Anesthesiologists (ASA) physical status I-III patients, aged 16–75 years, undergoing lower extremity orthopedic surgery with postoperative moderate to severe pain (VAS > 4) were consecutively enrolled. Patients with severe sweating, addiction to opioids, simultaneous injury, pain or surgery in upper extremities or major complications in the recovery room such as significant bleeding or hypotension were excluded. The study was approved by the regional ethics committee, and written informed consent was obtained from all patients.

### 3.2. Study Design

The surgeries were performed under general anesthesia or neuraxial block based on the decision of attending anesthetist. The general anesthesia was induced with fentanyl, thiopental sodium and atracurium and maintained with propofol and atracurium. The epidural and spinal anesthesia were performed with lidocaine and bupivacaine, respectively. In the recovery room, the patients' pain scores were assessed and in patients with VAS > 4, the ER between Li4 and Li11 acupuncture points were measured prior and following analgesic administration. The analgesic used was meperidine 0.3mg.kg-1. If the pain intensity was not reduced to VAS < 4, the meperidine dose was repeated. Following 20 minutes of the initial dose of analgesic, the ER of the acupoints as well as pain intensity was measured once more. Demographic variables, administered dose of meperidine and complications of treatment were recorded for all patients. Pain intensity was measured with a VAS, a 100-mm horizontal line with anchors of no pain and worst possible pain. Sedation score of the patients were assessed with a 5-point liker scale from 0 = agitate to 4 = unresponsive to verbal stimuli.

### 3.3. Measurement of Electrical Resistance

To determine the location of Li4 acupuncture point the patients were asked to oppose their right thumb against their pointing finger. The prominence in the muscle near to the crease between first and second fingers was considered as Li4 acupoint (22). To localize the Li11 acupoint, the patients were asked to flex their right elbow. The extremity of horizontal crease near to the lateral epicondyle points to the Li11 acupuncture point (22). In each acupuncture point a 13 mm steel needle (copper grip) with 0.25 mm diameter (Huan Qiu, China) was entered into the skin. The needle grip was insulated from skin contact using a sterile plastic washer containing a 1 mm hole. The needles were connected to the multimeter (My65, Mastech Company, Taiwan) with clips. Following connecting the needles with the multimeter, the clip, wires and multimeter were not moved throughout the assessment, and the patients were asked not to move their arms. Electrical resistance at acupuncture points were measured prior and 20 minutes following meperidine administration. Since each measurement was changed in time we record the maximum number of measurements in 30 second increments.

### 3.4. Statistical Analysis

Data are presented as mean (standard deviation) and frequency (percentage). Electrical resistance prior and following analgesic administration was compared with a paired t-test. The association between changes in ER and pain intensity was evaluated with the Pearson correlation coefficient. The change in ER was compared among different anesthesia methods with One-way ANOVA. All the comparisons were two tailed. P values < 0.05 were considered statistically significant. Statistical analysis was performed with SPSS version 11.5 (SPSS Inc., Chicago, IL).

## 4. Results

Of 50 enrolled patients, 35 were men and 15 were women. Using sedation scale, only 10% of subjects were agitated following arriving at the recovery room was recovered in the following assessment. None of the patients were unresponsive to verbal stimuli. The mean administered meperidine dose was 22.2 ± 4.8 mg. Nausea was recorded in five patients and pruritis in one subject. Following meperidine use, the mean VAS significantly decreased and the ER between the two acupoints was significantly increased (Table 1). In all but three patients the ER was increased following pain relief. However, Pearson correlation analysis did not reveal any association between the trends of pain intensity and ER (P > 0.05). Further analysis revealed the ER between the two acupoints was correlated with the weight of the patients (r = 0.37, P < 0.05). Interestingly, the ER change in patients operated under epidural anesthesia was significantly less than those who experienced general or spinal anesthesia whereas their pain intensity was not statistically different. When ER changes were adjusted for the weight of patients the difference was even more marked (P < 0.05) (Table 1).

**Table 1:** Outcome Measurements Prior and Following Meperidine Administration in Different Anesthesia Methods.

	General Anesthesia, (n = 23), Mean ± SD	Spinal Anesthesia, (n = 18), Mean ± SD	Epidural Anesthesia, (n = 9), Mean ± SD	Total, (n = 50), Mean ± SD
**VAS prior meperidine**	9.1 ± 1.3	8.2 ± 2.1	8.0 ± 1.8	8.5 ± 1.4
**VAS following meperidine**	2.6 ± 2.3	2.1 ± 1.7	1.8 ± 1.4	2.3 ± 2.0
**ER difference**	0.45 ± 0.55	0.56 ± 0.52	0.14 ± 0.16	0.43 ± 0.51
**ER adjusted** a	0.69 ± 0.81	0.83 ± 0.71	0.21 ± 0.23	0.65 ± 0.720
**Sedation, Median (quartile)**	2.1 ± 2.5	1.5 ± 1.2	1.5 ± 1.2	1.5 ± 1.2

Abbreviation: ER, Electrical resistance

^a^Electrical resistance adjusted to weight of patients

## 5. Discussion

Electrophysiologic studies of acupuncture points have been conducted from 1950s in the west with conflicting results up to the present time. Controversial findings may be due to the use of different acupoints, unlike instruments and the variable medical conditions of subjects. Several instruments have been suggested for measuring electrical skin resistance in acupuncture points. For instance in 2006 a new device including a field of 64 electrodes on a surface was presented for standardized measuring of electrical skin resistance. The authors claim that this device would eliminate the impact of various confounders such as pressure, angle of measurement, humidity of the skin and temperature in the measuring room (23). Another study has suggested that applying alternating current (AC) with a frequency of 20 to 100 Htz provides the optimum circumstance in measurement of electrical skin resistance (8). A review has stated that for measurement purposes, electrical skin resistance is not reliable and invasive electrodes may be required (15). To overcome these sources of controversy, we decided to measure the electrical resistance in a meridian instead of considering resistance at separate acupuncture points. Respecting the changes in the resistance instead of focus on absolute values may also eliminate the impact of some technical confounders. The measured ER varies in different studies. An earlier study reported that the ER in the center of the acupuncture points is about 10 K Ωand in surrounding areas 3 M Ω (12) however another survey demonstrated that the ER in different acupoints is variable. The related value for Li4 point in the right hand was reported to be 59 K Ω (11). In the present study, the ER resistance between Li4 and Li11 acupoints prior opioid use was 11.8 m Ωwhich is largely different from earlier reports of Li4 ER. The difference could be explained with the approximately 30 cm distance between Li4 distal to the wrist and Li11 in the elbow. In other words, we have evaluated the ER in a part of meridian instead of an isolated acupuncture point. This measurement strategy may eliminate the technical concerns raised from controversies on the diameter of acupuncture points. In the present study, pain relief and increase in the ER of the respected meridian were simultaneously observed in most of patients. However, no association was found between the trends of pain intensity and ER. Potential limitations in the study protocol such as considering one-sided Li4 measurements for lower extremity surgery in both genders might make the results challengeable. Measurements in more acupuncture points and possibly in the contralateral side may yield more reliable results. Data regarding the predictors of ER at acupuncture points in surgical conditions are rare. An earlier study has reported that encephaloma will reduce the ER in some specific acupoints (19). Another study tried to detect ear acupuncture points by measuring the electrical skin resistance under general anesthesia in patients undergoing orthopedic surgery. The authors claim that the frequently found patterns of ear acupoints with lower skin resistance in patients during orthopedic surgery can be useful for treatment of preoperative anxiety and postoperative pain relief (21). In this study, the changes in the ER of respected meridian following appropriate pain relief was significantly lower in patients experienced epidural anesthesia than either spinal or general anesthesia. The difference may be explained by the central stimulatory effect of lidocaine that reduces the control of stress response (24). Interestingly it has been proposed that both spinal and general anesthesia are effective in blocking the afferent limb of the sensory pathway but epidural anesthesia may not be reliably effective for this purpose (25, 26). Collectively, this evidence may reasonably suggest that ER at acupuncture points may predict some physiologic parameters such as stress response or autonomic function more reliably than pain. This hypothesis requires validation in further studies. If so, the measurement of ER at acupoints will gain several implications in daily clinical practice. In conclusion, coincidence of pain relief and change in the ER of acupuncture meridians without significant association leads us to a vague relationship between perception and electrophysiologic measurements. The disparity observed in different anesthesia methods may make the future pathway of electrophysiologic studies of acupuncture points more challenging and concurrently practical. However, the diagnostic value of electrical skin resistance for pain, stress response or any other physiologic outcome needs to be investigated in clinical trials with a well-defined control group, with more accurate instruments and possibly in different acupuncture meridians.
